# Dual role of Japanese encephalitis virus fusion loop peptide antibodies in Zika virus infection

**DOI:** 10.1371/journal.pntd.0014296

**Published:** 2026-05-04

**Authors:** Kexin Xi, Xuenan Zhang, Jingshu Li, Yuqi Zhao, Xiaoting Xie, Chenguang Shen, Bao Zhang, Li Zhu, Weiwei Xiao, Chengsong Wan, Yifan Lin, Linqing Wang, Yingfang Liu, Yuyan Wang, Jianhai Yu, Qinghua Wu, Wei Zhao

**Affiliations:** 1 Department of Biosafety Level 3 Laboratory (Guangdong), Guangdong Provincial Key Laboratory of Tropical Disease Research, Key Laboratory of Infectious Diseases Research in South China, School of Public Health, Southern Medical University, Guangzhou, Guangdong, China; 2 Department of Geriatrics, Nanfang Hospital, Southern Medical University, Guangzhou, Guangdong, China; Colorado State University, UNITED STATES OF AMERICA

## Abstract

Zika virus (ZIKV) is a key member of the *Flavivirus* genus that has emerged as a major global public health concern. The fusion loop region (residues 98–110), located within domain II of the envelope protein, is highly conserved among flaviviruses, including ZIKV and Japanese encephalitis virus (JEV). However, the functional consequences of such conservation for cross-reactive immunity remains unclear. Here, we integrated bioinformatic analyses, functional assays *in vitro* and mouse models *in vivo* to systematically determine the effects of antibodies directed against the JEV fusion loop (FL) region on ZIKV infection. Sequence alignment and structural analysis revealed complete amino acid identity and almost identical three-dimensional conformations between the FL regions of the two viruses, providing a molecular basis for cross-reactivity. Antisera generated against the JEV FL region recognized ZIKV particles and displayed concentration-dependent bidirectional effects. Increased and decreased antibody levels respectively neutralized viral entry and replication, and facilitated infection *via* antibody-dependent enhancement (ADE). These effects were confirmed *in vivo*, in which high and low antibody doses reduced tissue pathology and improved survival, and increased viremia and exacerbated inflammatory responses, respectively. These findings highlight the importance of antibody concentration in determining whether cross-reactive responses to conserved structural elements engender neutralization or enhancement response. Our findings provide experimental evidence for assessing ZIKV susceptibility in JEV-vaccinated populations and offer structural insights for designing flavivirus vaccines that maximize protection while minimizing ADE risk. These findings further highlight potential pathogenic and clinical considerations for optimizing vaccine formulations to reduce cross-reactive enhancement risks.

## Author summary

Mosquito-borne flaviviruses such as Japanese encephalitis (JEV) and Zika (ZIKV) viruses induce antibodies that recognize related viruses. We investigated how antibodies produced after JEV vaccination might affect subsequent ZIKV infection. Our study focused on the fusion loop (FL) region of the envelope protein—a conserved segment present in both JEV and ZIKV. We found that this site was almost identical in both viruses. Laboratory findings showed that antibodies raised against the JEV FL region can also bind to ZIKV and exhibit concentration-dependent bidirectional effects. That is, high antibody levels block infection and protect the host, whereas low levels facilitate viral entry into cells and worsen disease. High antibody levels reduced viral load and inflammation, whereas low levels increased infection and tissue damage in mice. Our findings highlight the delicate balance between beneficial and detrimental antibody responses and help to explain why past vaccination or infection can sometimes fail to protect against, or even exacerbate infection with a related virus. As JEV and ZIKV co-circulate in many endemic regions, this mechanism is relevant for understanding real-world susceptibility. It also highlights the importance of evaluating vaccine-induced antibody profiles to ensure flavivirus vaccines are both safe and effective.

## Introduction

Zika virus (ZIKV) is a single-stranded, positive-sense RNA virus transmitted by *Aedes* mosquitoes. Zika virus belongs to the *Flaviviridae* family and is closely related to dengue virus (DENV), Japanese encephalitis virus (JEV), and yellow fever virus (YFV) [1]. Since its initial discovery in the Zika Forest of Uganda in 1947, ZIKV has caused outbreaks in Africa, Asia, the Americas and Oceania [2]. The first large-scale outbreak occurred on Yap Island in 2007, followed by widespread transmission across the Americas in 2015. The World Health Organization declared ZIKV a “Public Health Emergency of International Concern” during 2016 [3]. By 2019, ZIKV transmission had spread over 80 countries and regions worldwide highlighting its persistent global health threat [4–6]. Imported outbreaks have recently occurred in China, underscoring the need for continued surveillance [7].

The ZIKV genome is ~ 11 kb in length and encodes a single polyprotein that is processed into the structural capsid protein, pre-membrane glycoprotein, and glycosylated E protein, and seven non-structural proteins (NS1, NS2A, NS2B, NS3, NS4A, NS4B, and NS5) [8]. Among these, the envelope (E) protein is instrumental in mediating viral entry and inducing immune responses, serving as the primary target of most antibody responses [9,10]. The E protein is highly conserved among flaviviruses, and the fusion loop region (residues 98–110) within domain II (EDII) represents a structurally conserved segment. The FL region is an important structural element of the E protein, critical for viral membrane fusion and potentially mediating cross-reactive antibody responses [11]. Although considerable research has focused on cross-reactive antibodies targeting DII, the FL region has also been identified as a highly conserved segment whose functional role in protective immunity and pathogenesis remains to be fully elucidated [12].

Within the flavivirus immune response, this FL region represents a structurally conserved segment within EDII of the E protein. It can mediate neutralization [13] and exacerbate infection through antibody-dependent enhancement (ADE) *via* the fragment crystallizable gamma receptor (FcγR) pathway [14]. Epidemiological studies have reported increased disease severity in secondary DENV infections [15], consistent with the ADE observed *in vitro*, highlighting the potential relevance of cross-reactive flavivirus antibodies in human populations [16]. Structural modification or reduced exposure of the FL region in the ZIKV envelope protein significantly decreases ADE during dengue virus infection, suggesting that cross-reactive antibodies predominantly target this conserved region [17]. Furthermore, antibodies targeting the FL region are generally weakly neutralizing but have been reported to confer partial protection *in vivo* [13]. These observations highlight the dual functional nature of FL-directed flavivirus antibodies. Serological studies have revealed 56.1% sequence homology in the E proteins between JEV and ZIKV [18]. Although this suggests that shared regions, such as the FL region, are involved in immune interactions, the conclusions remain tentative. Considering the widespread application of JEV vaccines worldwide, the possibility that antibodies induced by JEV vaccination targeting the FL region may enhance ZIKV infection under certain conditions has significant public health implications. Although antibodies generated after JEV vaccination or infection have some cross-binding capacity against ZIKV [19,20], the functional outcomes of antibodies targeting the JEV FL region during ZIKV infection, including neutralization and ADE, remain incompletely understood and are likely context-dependent [21]. Therefore, the functional effects of antibodies induced by JEV vaccination or infection that target the FL region on ZIKV infection warrant systematic investigation. This would help to elucidate cross-immunity mechanisms within the *Flavivirus* genus and holds practical significance for assessing potential risks in vaccinated populations, optimizing vaccine design, and developing new antibody therapy strategies.

Accordingly, we focused on the FL region of the JEV E protein. We generated mouse polyclonal antibodies (pAbs) and systematically evaluated their effects on ZIKV infection *in vitro* and *in vivo*. We elucidated the immunological role of antibodies recognizing the FL region in cross-flavivirus interactions and provided mechanistic insights for vaccine optimization and antibody-based interventions by assessing concentration-dependent neutralization and enhancement.

## Results

### Conservation and antigenicity of the fusion loop region in JEV and ZIKV

**Fig 1:**
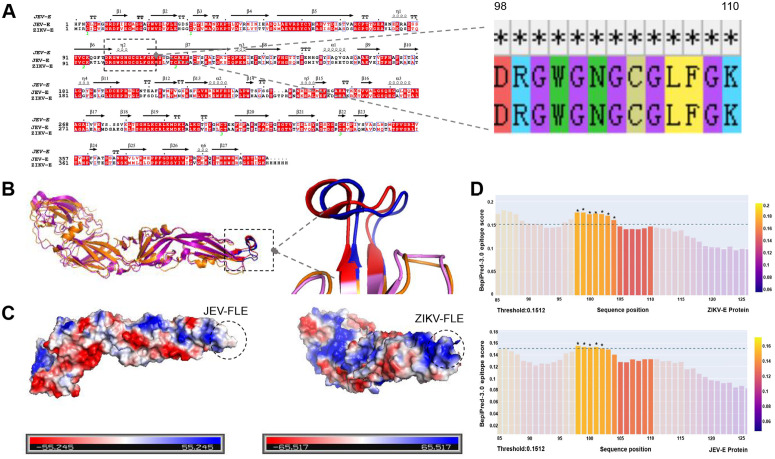
Comparative analysis of the fusion loop region in JEV and ZIKV E proteins. (A) Sequence alignment of the FL region core region, showing complete conservation between JEV and ZIKV. Colors indicate amino acid types. (B) Tertiary structure superposition of the FL region, with a Cα RMSD of 0.247 Å, indicating high structural similarity. Orange/red: ZIKV protein/FP loop; purple/blue: JEV protein/FP loop. (C) Surface electrostatic potential of the FL region. ZIKV exhibits a positively charged surface (blue), while JEV is neutral (white). Color scale: blue, positive; white, neutral; red, negative. (D) Linear B-cell epitope prediction. ZIKV FL region forms a strong epitope of seven residues (98–104), whereas JEV comprises five residues (98–102), indicating stronger predicted antigenicity in ZIKV(* indicates > 0.01512).

We systematically analyzed E proteins using a bioinformatic approach to determine the potential of JEV and ZIKV cross-reactivity. Sequence alignment revealed that the core region of the FL is 100% identical between JEV and ZIKV (residues 98–110; Fig 1A), providing a fundamental molecular basis for potential cross-reactive antibody recognition. We then examined the structural conservation of this region *via* tertiary structure superposition, which revealed overlapping FL regions of the two viruses in three-dimensional space, with a Cα root-mean-square deviation (RMSD) of only 0.247 Å (Fig 1B). These findings indicated almost identical spatial conformations. However, sequence and structural conservation alone are insufficient to predict cross-reactivity. We analyzed the surface electrostatic potential of the FL to further explore the mechanistic basis of antibody binding. The results showed that the epitope in ZIKV presents a positively charged surface, whereas the corresponding region in JEV is electrostatically neutral (Fig 1C). This difference suggested that antibodies are preferentially attracted to and bind the positively charged ZIKV surface, providing a structural and physicochemical explanation for the stronger predicted antigenicity of the ZIKV FL. We also evaluated the immunogenic potential of the FL in each viral context by linear B-cell epitope prediction. While we predicted that the FL of both viruses was immunoreactive, the signal strength differed. The FL region in ZIKV spans seven consecutive residues (98–104) with BepiPred-3.0 scores exceeding 0.01512, whereas that in JEV comprises five consecutive residues (98–102) exceeding the same threshold (Fig 1D). These results indicate that the FL region may be more immunodominant in ZIKV than in JEV.

### Cross-reactivity and concentration-dependent functional effect of antibodies against the JEV fusion loop region with ZIKV

**Fig 2:**
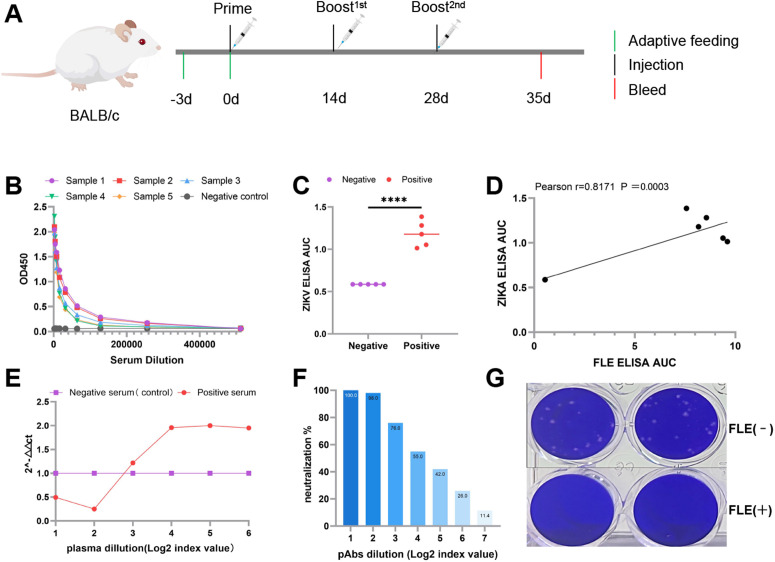
Cross-reactivity and concentration-dependent functional effects of anti-JEV fusion loop antibodies *in vitro.* The BALB/c mouse image (https://scidraw.io/drawing/799) and syringe image (https://scidraw.io/drawing/385) were obtained from SciDraw and are licensed under the Creative Commons Attribution 4.0 International (CCBY 4.0) license. (A) Schematic of the mouse immunization and experimental workflow. (B) ELISA titers of mouse sera against the JEV FL peptide, showing successful immunization. (C) Comparison of cross-reactivity (area under the curve [AUC]) between five JEV FL-positive and five negative sera with ZIKV virions (*p* < 0.0001). The AUC was calculated using GraphPad Prism to quantify the overall binding response across the dilution series; all measurements were performed at OD_450_. (D) Correlation between anti-JEV FL antibody titers and ZIKV cross-reactivity.(E) Antibody-dependent enhancement (ADE) in K562 cells at different serum dilutions, measured by relative ZIKV RNA levels. (F) Plaque reduction neutralization percentage in Vero cells at different serum dilutions. (G) Representative plaque assays of ZIKV in Vero cells treated with undiluted JEV FL-negative (top) or positive (bottom) sera. All experiments were independently repeated three times.

We validated the bioinformatic predictions and assessed functional consequences using mouse immunization and *in vitro* assays (Fig 2A). Enzyme-linked immunosorbent assays (ELISA) revealed that immunized mouse sera reached a titer of 10⁵ against the JEV FL peptide, confirming successful immunization (Fig 2B).We then evaluated the cross-reactivity of these sera with ZIKV virions, and determined that five JEV FL-positive sera bound to ZIKV with significantly higher affinity compared with those of negative controls (*p* < 0.0001; Fig 2C). Antibody titers showed a positive correlation trend with ZIKV binding (Pearson r = 0.8171; Fig 2D), confirming cross-reactive recognition. We then examined the functional effects of these antibodies on ZIKV infection, and analyzed K562 cells, which are used to assess ADE, using qRT-PCR. Relative to the negative control, viral RNA expression increased significantly at plasma dilutions of 2^4^–2^6^, with 2^(-ΔΔCt)^ values of 1.96, 2.00, and 1.95 at dilutions of 2^4^, 2^5^, and 2^6^, respectively (Fig 2E). This approximately two-fold increase in viral RNA indicates prominent ADE within this dilution range. Neutralization assays in Vero cells revealed a gradual decline in neutralization ratios (%) with Abs serum dilution increased in log₂ increments. Neutralization was substantial at dilutions of 2^1^–2^3^, with plaque reduction > 50% and a plaque reduction neutralization test (PRNT₅₀) of 1:32, whereas further dilution almost abolished neutralization (Fig 2F). Therefore, the high concentrations of antibodies confer neutralizing protection, whereas low concentrations result in loss of neutralization activity and trigger ADE. These results were consistent with the findings in K562 cells. Additionally, compared with controls, ZIKV plaque formation was significantly reduced in Vero cells incubated with JEV FL-positive sera (Fig 2G).

### Dual role of FL antibodies in ZIKV infection confirmed *in vivo*

**Fig 3:**
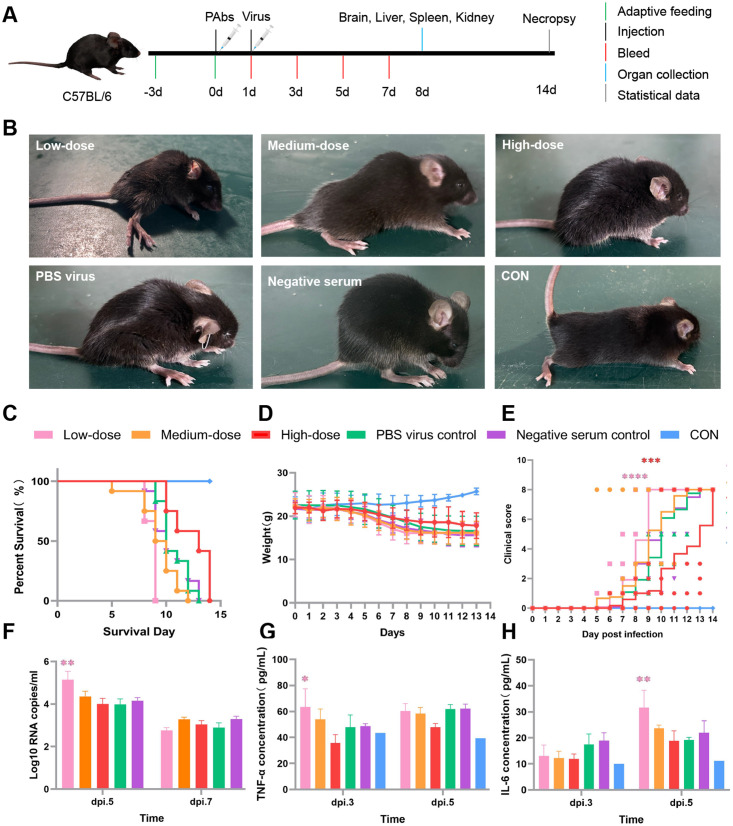
Concentration-dependent protective and ADE effects of anti-JEV FL antibodies *in vivo.* The C57BL/6 mouse image was obtained from Wikimedia Commons (https://commons.wikimedia.org/wiki/File:201707_mouse_3D_black.svg) and is licensed under CCBY 4.0. The syringe image was sourced from SciDraw (https://scidraw.io/drawing/385) and is also licensed under CCBY 4.0. The mouse symptom photograph was taken by the authors. (A) Schematic of the passive immunization and ZIKV challenge experimental design.(B) Clinical symptoms on day 8 post-infection in each group.(C) Kaplan–Meier survival curves of mice.(D) Body weight changes over the course of infection.(E) Clinical disease scores of infected mice.(F) Viral loads in blood at different time points post-infection, measured by qRT-PCR. Statistical significance at dpi 5 compared with virus-only controls was determined by t-test.(G) Serum TNF-α concentrations in each group.(H) Serum IL-6 concentrations in each group. Data were analyzed using two independent-sample t-tests;ns, not significant; *p < 0.05, **p < 0.01, ***p < 0.001,****p < 0.0001. (* indicates statistical significance compared with the PBS control. The color of * corresponds to the color of its respective treatment group in the figure).

We established a passive immunization mouse model to validate the dual effects of anti-JEV FL antibodies on ZIKV infection *in vivo* (Fig 3A). Six- to nine-week-old double-knockout mice on a C57BL/6 background were used in this study. The mice were originally generated by and purchased from Shanghai Model Organisms Center, Inc. (Shanghai, China), and were subsequently bred and maintained under specific pathogen-free (SPF) conditions at Cyagen Biosciences (Suzhou, China). High-, medium-, and low-dose groups (n = 12 each) received 2^3^, 2^5^, and 2^7^ dilutions of pAbs, respectively. Based on *in vitro* neutralization and ADE assays, which showed maximal neutralizing effects at antibody dilutions of 2^1^–2^3^ and ADE effects at lower concentrations—we selected three representative dilutions for *in vivo* evaluation: 2^3^ (high dose), 2^5^(medium dose), and 2^7^ (low dose), to capture both protective and potentiating effects. The mice were challenged 24 h later with 10³ plaque-forming units (PFU) of ZIKV injected intraperitoneally (i.p.). Mice with negative serum and virus-only served as controls. The antibodies exhibited a dose-dependent biphasic effect (Fig 3B). The high-dose group showed improved outcomes, with the longest survival (life extension rate +15.63%; Table 1), lowest clinical scores throughout the infection course, with significant differences observed from post-infection day (PID) 9 onward (*p* < 0.001 vs PBS control) and minimal body weight loss. These results indicated that a weak neutralizing protective effect mitigated disease progression.

**Table 1:** Median survival time and life extension rate in each group of mice.

Group	n	Survival Count	Death Count	Median^a^ Survival	Survival Days^b^ (mean ± SD)	Life Extension^c^ Rate(mean ± SD)%
**Low-dose**	12	0	12	9	8.67 ± 0.49	-18.75% ± 4.62%**
**Medium-dose**	12	0	12	9	9.23 ± 1.79	-13.46% ± 16.75%*
**High-dose**	12	0	12	13	12.33 ± 1.78	15.63% ± 16.64%*
**Negative serum control**	12	0	12	10	10.42 ± 1.73	-2.34% ± 16.22%
**PBS virus control**	12	0	12	10	10.67 ± 1.30	0
**Blank control**	5	5	0			

**JEV Japanese encephalitis virus, ZIKV, Fusion loop peptide, Polyclonal antibodies**

^a^Median survival of mice in each group

^b^Survival days (mean ± SD) of mice in each group

^c^Life extension rate (mean ± SD) of mice in each group

We performed Student’s t-tests to compare each group with the PBS control, with significance denoted as *p < 0.05, **p < 0.01, and ***p < 0.001.

In contrast, the low-dose group exhibited typical ADE, namely a steep decline in survival (life extension rate, −18.75%), the most rapid and pronounced increase in clinical scores from PID 8 onward (*p* < 0.0001 vs control), dramatic body weight loss, and severe neurological symptoms of limb and systemic paralysis, ultimately leading to death (Fig 3C–3E). These results indicated that low-dose antibodies did not confer protection, significantly exacerbated pathological damage, and accelerated mortality. The group with the medium dose responded with intermediate responses in some parameters, but survival and weight changes were closer to those in the low-dose group, with limited protection. To further assess the impact of anti-JEV FL antibodies on ZIKV replication, we assessed viral RNA levels in blood at several time points after infection. At PID 5, the low-dose group had significantly higher viral RNA levels than the virus-only control, indicating ADE; thus, low-dose antibodies were not protective, confirming the concentration-dependent effects of antibodies (Fig 3F). Analysis of inflammatory responses further revealed the immunopathological mechanisms of ADE. At PID 3, levels of the pro-inflammatory cytokine tumor necrosis factor alpha (TNF-α) were significantly elevated in the low-dose (63.44 ± 13.97 pg/mL) and medium-dose (54.10 ± 7.86 pg/mL) groups compared to that of the PBS virus control (47.99 ± 9.33 pg/mL), with the increase being the greatest in the low-dose group. By PID 5, levels of the key inflammatory cytokine interleukin (IL)-6 remained significantly higher in the low-dose group (31.64 ± 6.69 pg/mL) than in the PBS virus control group (19.15 ± 1.09 pg/mL; Fig 3G). This indicated that ADE induced a pronounced and sustained systemic inflammatory response that probably contributed to exacerbated pathology and accelerated mortality.

### Histopathological analysis

**Fig 4:**
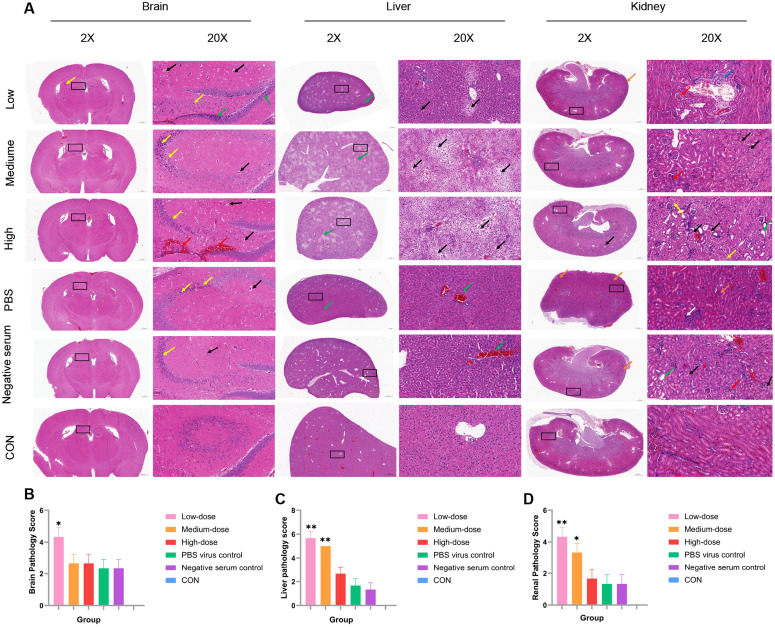
Histopathological alterations and semi-quantitative lesion scoring of major organs in mice. (A) Representative H&E-stained sections of brain, liver, and kidney tissues (the black box indicates the magnified field of view).Brain (hippocampal region): black arrows indicate perivascular edema; yellow arrows indicate pyramidal neuron nuclear shrinkage; red arrows indicate focal hemorrhage in the DG region; green arrows indicate nuclear shrinkage of granular cells in the DG region.Liver: black arrows indicate hepatocellular steatosis; green arrows indicate vascular congestion.Kidney: black arrows indicate tubular epithelial swelling; yellow arrows indicate vacuolar degeneration; red/white arrows indicate glomerular lesions; green arrows indicate tubular dilation; brown arrows indicate interstitial congestion; blue arrows indicate inflammatory (lymphocytic) infiltration. (B) Semi-quantitative composite pathological score of brain lesions. (C) Semi-quantitative composite pathological score of liver lesions. (D) Semi-quantitative composite pathological score of kidney lesions.Pathological grading was performed according to the INHAND (International Harmonization of Nomenclature and Diagnostic Criteria for Lesions in Rats and Mice) four-tier scoring system. Data were analyzed using two independent-sample t-tests; ns, not significant; *p < 0.05, **p < 0.01, ***p < 0.001.

We collected brain, liver, kidney, and spleen tissues from the mice in all groups for histopathological examination before death. Spleen tissues did not significantly differ among the groups (S1 Fig), whereas pathological changes were distinct in the brain, liver, and kidneys (Fig 4A).

Histopathological analysis of the brain revealed nuclear pyknosis of pyramidal neurons in the CA3 region in all groups (yellow arrows). This was characterized by reduced cellular volume, irregular nuclear morphology, indistinct nucleocytoplasmic boundaries, and increased staining intensity. Such changes were sporadic in the low-dose group and were accompanied by focal hemorrhage in the dentate gyrus (DG) region (red arrows) and perivascular edema (black arrows). The low-dose group developed very severe lesions (clinical score > 4), whereas the other groups exhibited significant lesion aggravation (score > 2). The pathological score of this group was elevated compared with that of the PBS control (Fig 4B). The medium-dose group had a few pyknotic pyramidal neurons, with more pronounced interstitial edema in the DG region. Lesions in the high-dose group extended to the CA3–CA4 regions, nuclear pyknosis was evident in granule cells in the DG region (green arrows), and interstitial edema was widespread. The PBS and negative-serum controls had mild nuclear pyknosis within the CA2–CA3 regions and obvious interstitial edema but no inflammatory cell infiltration. In contrast, the blank control group has intact hippocampal architecture with normal cellular morphology and an orderly neuronal arrangement.

Histopathological analysis of the liver revealed various degrees of hepatocyte steatosis (black arrows) manifesting as round cytoplasmic vacuoles in all experimental groups. Extensive steatosis was accompanied by disorganized hepatic plates low- and medium dose groups. The low-dose and medium-dose groups exhibited very severe lesions (clinical score > 4), and the high-dose group showed significant lesions (score > 2), with correspondingly higher pathological scores than the PBS control (Fig 4C). In contrast, the PBS and negative control groups displayed only mild lesions. Although the high-dose group had multifocal steatosis, its hepatic plates remained relatively well organized and sinusoidal structures were preserved. The structural characteristics of the PBS and negative-serum groups were similar, with rounded hepatocytes, orderly hepatic plates, and mild vascular congestion (green arrows). No histopathological abnormalities were found in the blank control group.

Histopathological analysis of kidney tissues revealed extensive pathological changes in the low-dose group, including tubular epithelial cell edema (black arrows), vacuolar degeneration (yellow arrows), and occasional tubular dilation (green arrows). The medium-dose group had mild glomerular capillary dilation and congestion (red arrows), along with more prominent tubular epithelial edema. The low-dose group developed very severe lesions (clinical score > 4), the medium-dose group showed significant lesions (score > 3), whereas lesions in the other groups were mild. The pathological scores were higher for the low- and mid-dose groups compared with the PBS control, with changes being more pronounced in the low-dose group (Fig 4D). The high-dose group exhibited largely normal tubular epithelial morphology, with sporadic lymphocytic infiltration (blue arrows) and vascular congestion (orange arrows) within the interstitium. The PBS group had irregular renal morphology, characterized by glomerular hypercellularity (white arrows) and interstitial vascular congestion. The negative-serum group had comparatively mild lesions, consisting mainly of glomerular capillary congestion and limited tubular edema. The blank controls had intact renal architecture with normal morphology and no detectable pathological changes.

## Discussion

The present findings revealed high conservation of the FL within E protein of JEV and ZIKV, as well as its dual functional role in cross-immunity. Sequence and structural analyses uncovered that the FL regions of the two viruses were identical at the amino acid level and almost completely superimposable in a three-dimensional conformation (RMSD = 0.247 Å). These findings provide a molecular basis for the cross-reactivity that is common among flaviviruses [11, 18]. Despite this structural conservation, we identified distinct electrostatic features. The FL surface of ZIKV exhibited a more positively charged electrostatic potential and a stronger B-cell epitope signal. This suggests greater epitope exposure and immunodominance, which may enhance antibody recognition. These subtle charge differences likely influenced antibody binding orientation and epitope accessibility, thus explaining the high-affinity cross-reactivity of ZIKV by anti-JEV-FL antibodies and their complex functional outcomes.

Functionally, anti-JEV-FL antibodies exhibited a characteristic concentration-dependent dual effect. At high concentrations, the antibodies did not reduce viral RNA levels but suppressed inflammatory responses and alleviated disease progression. In contrast, they significantly enhanced viral replication and inflammation at low concentrations, resulting in a typical ADE effect. This bidirectional phenomenon highlights the delicate balance between neutralization and enhancement, jointly governed by antibody concentration and FcγR-mediated signaling [22–24]. Our data suggest that FL-targeting cross-reactive antibodies mediates protective or pathogenic outcomes depending on their concentration, although further studies with larger sample sizes are needed to confirm these findings. This is a novel mechanistic insight into flavivirus cross-immunity. Mechanistically, the ADE at low antibody levels likely results from FcγR-dependent internalization of antibody-virus complexes [14]. At sufficient antibody concentrations, virions are fully neutralized and unable to bind host receptors. However, at sub-neutralizing levels, partially coated virions can enter FcγR-expressing monocytes, leading to productive infection. This “antibody threshold model” explains a nonlinear relationship between antibody titers and disease severity, emphasizing the antibody quantity rather than specificity alone as a critical determinant of the immune outcome. The results *in vivo* further validated these mechanisms. The mice treated with a high dose of antibody had prolonged survival and reduced pathological damage, whereas more severe lesions were accompanied by a rising viral load and an increased inflammatory response in the low-dose group. The FL might primarily act by augmenting the host immune response. Although it has some neutralizing activity that contributes to viral load reduction and symptom attenuation, its ability to fully prevent severe disease progression might be limited. In addition, TNF-α and IL-6 levels were significantly elevated in the low-dose group, suggesting that Fc-mediated viral invasion in conjunction with aberrant immune activation driving pathological damage. Taken together, these data link structural conservation to immune function outcomes, emphasizing the central role of the FL in flavivirus cross-immunity and immunopathology.

These results have significant implications for vaccine design and public health. Considering the widespread use of JEV vaccines worldwide [25], FL-specific antibodies might influence ZIKV infection outcomes in regions with co-circulation. High-titer antibodies might confer partial protection, whereas sub-neutralizing titers could conversely increase the risk of enhanced infection. Therefore, the design of next-generation pan-flavivirus vaccines should carefully address conserved cross-reactive regions such as the FL, possibly through structural modification or masking, to maintain immunogenicity while minimizing ADE risk [26]. This strategy aligns with recent dengue and ZIKV vaccine findings showing that rational FL remodeling can reduce enhancement without compromising neutralization [27]. Moreover, our findings highlight the need to quantify antibody titers and Fc receptors when evaluating immune protection. Antibody specificity might yield opposing outcomes depending on concentration and receptor engagement. In regions where flaviviruses are co-endemic, preexisting or vaccine-induced antibodies should, therefore, be considered potential double-edged swords that can mediate either cross-protection or enhanced disease depending on immune conditions.

This study has several limitations. We used murine polyclonal sera, which might contain non-FL antibodies, and differences between murine and human FcγR systems could affect ADE potency [28, 29]. In addition, the results *in vivo* were derived from a single susceptible mouse model (6–9-week-old C57BL/6 mice; n = 12 per group), Thus, their statistical power and biological generalizability are constrained, and they should be interpreted as preliminary *in vivo* validation rather than definitive efficacy evidence. As these observations are preliminary, their direct public health implications in naturally exposed or vaccinated populations are incompletely established and require confirmation through population-level serological and clinical studies. Importantly, future studies comparing sera from JEV-vaccinated or JE-immune individuals across different vaccine platforms, particularly in regions where multiple flaviviruses co-circulate, will be needed to determine whether vaccine-induced antibody profiles differentially influence ZIKV neutralization and ADE risk. To overcome the limitations of using pAbs, we generated several FL-specific monoclonal antibodies (mAbs) from immunized mice and conducted an initial functional assessment. Notably, at least one of these mAbs may exhibited a significant ADE effect *in vitro*. Future studies combining these mAbs with larger-scale animal models will be essential to further elucidate the specific roles of FL in immune protection and ADE. Moreover, immunization with non-fusion loop peptides could serve as a useful negative control to further assess epitope specificity. In addition to the further examination of the molecular determinants of neutralization and ADE through cryo-electron microscopy structural analysis and population serological studies. Quantitative modeling combining antibody kinetics and receptor signaling pathways will help to predict ADE thresholds in clinical settings [30].

## Materials and methods

### Ethics statement

Animal experiments were approved by the Ethical Committee for Animal Research of Southern Medical University (Permit number: SMUL202309008) and conducted based on the guidelines of the Ministry of Science and Technology of China.

### Serum samples and antibody preparation

The synthetic peptide corresponding to the FL (amino acid sequence: DRGWGNGCGLFGK) [31] of the JEV E protein was synthesized and conjugated to keyhole limpet hemocyanin (Megathura crenulata, Cat# 374805, Sigma-Aldrich, St. Louis, MO, USA) by GL Biochem (Shanghai, China). Peptide purity (> 98%) was confirmed using reverse-phase high-performance liquid chromatography, and molecular weight was verified by electrospray ionization mass spectrometry. Mouse pAbs against the JEV FL were obtained through peptide immunization. For each mouse, 100 μg of JEV FL peptide was emulsified in an equal volume (100 μL) of Freund’s adjuvant (Sigma-Aldrich) to form a stable water-in-oil emulsion prior to immunization. For the negative control group, mice received an equal volume of Freund’s adjuvant emulsified with PBS without peptide antigen. Age-matched female BALB/c mice (6–8-weeks-old; n = 5 per group; provided by the Animal Experiment Center of Southern Medical University, Guangdong, China) were randomly assigned to either the immunization or negative control (adjuvant only) group. Immunization was performed *via* intramuscular injection of the peptide emulsified in Freund’s adjuvant. The initial immunization used complete Freund’s adjuvant, followed by two booster immunizations at weeks 2 and 4 using incomplete Freund’s adjuvant [32]. Seven days after the final booster, whole blood was collected *via* orbital puncture, then serum was separated and stored at –80 °C. Antibody titers and antigen specificity were determined using ELISA, and sera with high antibody titers were selected and pooled for subsequent *in vivo* and *in vitro* experiments.

### Viruses and cells

We used the ZIKV strain Z16006 (KU955589.1; GenBank, National Center for Biotechnology Information, Bethesda, MD, USA) [33] for cross-reactivity assays, neutralization tests, and ADE studies. Vero (ATCC CCL-81) [34] and K562 (ATCC CCL-243) [35] cells were cultured in Dulbecco’s modified Eagle (DMEM) and RPMI-1640 media, respectively, supplemented with 10% heat-inactivated fetal bovine serum (Sigma-Aldrich, MO, USA). All cells were maintained at 37°C in a humidified incubator under a 5% CO₂ atmosphere.

### Bioinformatics analysis

Amino acid sequences and three-dimensional structures of the E proteins of JEV (strain SA-14; PDB: 5MV1) [36] and ZIKV (strain Z16006; PDB: 5JHM) [37] were retrieved from the RCSB Protein Data Bank. Multiple sequence alignments were generated using the ESPript 3.0 online tool. Structural superimposition of the two viral E proteins was conducted in PyMOL, with the FL regions highlighted for visualization. In this study, the fusion loop region (residues 98–110) within domain II was defined as the structural region of interest, and antibodies described herein refer to those targeting this region rather than a single discrete epitope. Electrostatic surface potential maps of the E protein surfaces were generated using the APBS Electrostatics plugin in PyMOL. B-cell linear epitopes were predicted using the BepiPred-3.0 online server with default parameters.

### ELISA antibody titration and cross-reactivity assay

ZIKV antigen (10³ PFU/well) and synthetic peptides (50 μg/well) were diluted in coating buffer (0.1 M sodium carbonate–bicarbonate, pH 9.4), added to 96-well plates (100 μL/well), and incubated overnight at 4 °C. After the plates were washed three times with PBS containing 0.1% Tween-20, non-specific protein binding was prevented by incubation with 5% skim milk blocking buffer (300 μL/well) for 4 h at room temperature. Heat-inactivated mouse serum was initially diluted 1:1000 in PBS, then serially diluted two-fold. Aliquots (100 μL/well) of each dilution were added to the coated plates. FL peptides -positive serum, FL peptides -negative serum, and PBS were used as positive, negative, and blank controls, respectively. After incubation for 1.5 h followed by washing, the plates were incubated with horseradish peroxidase-conjugated Affinipure goat anti-mouse IgG (H + L) secondary antibody (1:5000; Cat# SA00001–1, Proteintech, Rosemont, IL, USA) for 1 h at 37 °C. Signals were developed using p-phenylenediamine dihydrochloride substrate, stopped with sulfuric acid, and optical density was measured at 450 nm using a microplate reader [38].The area under the curve (AUC) was calculated using GraphPad Prism to quantify the overall binding response across the dilution series, with a higher AUC suggesting stronger overall cross-reactive binding.

### ADE assay with fusion loop immunized mouse serum

Mouse anti-JEV FL pAbs serum, derived from an equal-volume pool of high-titer sera from immunized BALB/c mice as determined by ELISA, was serially diluted two-fold in RPMI-1640 medium and incubated with equal volumes of ZIKV at 37 °C for 1 h. The antibody-virus mixtures were added to K562 cells (MOI = 0.5) and incubated for 2 h. The cells were then cultured in maintenance medium and incubated for 2 d under standard conditions. Total RNA was extracted and viral RNA levels were quantified by qRT-PCR targeting the ZIKV NS5 region [39] (forward: 5′-GGCRTTRGCCATCAGTCG-3′; reverse: 5′-ATGGAGCATCCGKGAGACT-3′). GAPDH was used as the internal reference (forward: 5′-CATCCTGGGCTACACTGAGC-3′; reverse: 5′-AAAGTGGTCGTTGAGGGCAA-3′) [40].

Relative viral RNA levels were calculated using the ΔΔCT method, and fold-change values are expressed as 2^⁻ΔΔCT^ [41].

### PRNT performed with mouse serum raised against the JEV fusion loop region

Anti-JEV FL mouse pAbs were serially diluted two-fold in DMEM, mixed with an equal volume of ZIKV (MOI = 0.5), and incubated for 1 h at 37 °C. The antibody-virus mixtures were then added to Vero cell monolayers in 24-well plates and incubated for 2 h at 37 °C. As a negative control, serum pooled from adjuvant-immunized mice (without peptide antigen) was used. After removing the inoculum, the cells were overlaid with 1.2% methylcellulose in maintenance medium to allow plaque formation. Following 5–7 d of incubation, the plates were fixed and stained with crystal violet for plaque visualization and counting. The percentage of plaque reduction was calculated using the following formula: Percentage plaque reduction = (Number of plaques in control wells − Number of plaques in treated wells)/Number of plaques in control wells × 100% [42].

### ZIKV challenge in interferon-deficient mice with anti-FL serum

Heat-inactivated anti-JEV FL mouse serum was serially diluted in PBS. A susceptible mouse model, comprising 6–9-week-old Ifnar1^−^/^−^Ifngr1^−^/^−^ C57BL/6 mice (n = 12 per group), received 200 µL of the pooled diluted serum *via* i.p. injection. The mice were originally obtained from Shanghai Model Organisms Center, Inc. and were bred and maintained under SPF conditions at Cyagen Biosciences. Twenty-four hours later, the mice were challenged i.p. with 10^4^PFU of ZIKV, followed by administration of high-, medium-, or low-dose FL serum diluted to 2^3^, 2^5^, and 2^7^, respectively. The control groups consisted of mice injected with serum from adjuvant-immunized mice (without antigen) and PBS-injected mice. Mice were monitored daily for 14 d for body weight changes, clinical signs, and survival. Clinical signs were scored as follows: 0, no obvious symptoms; 1, hunching and piloerection; 2, tremor and reduced activity; 3, motor impairment without paralysis; 4, single-limb paralysis; 5, double-limb paralysis; 6, triple-limb paralysis; 7, full paralysis; and 8, death. Survival was quantified using the Life Extension Rate, which was calculated for each group as follows: Life Extension Rate = [(Average Days in the Pre-Stored Antibody Dose Group - Average Days in the Virus Control Group)/ Average Days in the Virus Control Group] × 100%. Viral burden in serum was assessed by qRT-PCR targeting the ZIKV NS5 gene [43].

### ELISA detection of serum TNF-α and IL-6

Whole blood was collected by retro-orbital puncture and centrifuged to obtain serum samples that were diluted 1:10 and 1:5 with IL-6 and TNF-α with sample diluent (100 µL per well). Cytokines were quantified using mouse TNF-α and IL-6 ELISA kits (EMC102a.96 and EMC004.96, respectively; China Xinbosheng Biotechnology Co., Ltd., Shenzhen, China) as described by the manufacturer.

### Histopathological analysis

Brains, livers, kidneys, and spleens were collected at PID 8 from each group (n = 3) and fixed in 10% neutral-buffered formalin at 4 °C for 48 h [44]. The tissues were trimmed, dehydrated, embedded in paraffin, sectioned, and stained with hematoxylin and eosin according to the standard pathological testing procedures of Shinanke Biotechnology Ltd. The groups included: (i) CON, representing healthy mice with normal development; (ii) PBS control, receiving virus only; and (iii) negative serum control, receiving sera from mice immunized with adjuvant only (no antigen). The slides were subsequently examined under a light microscope.

### Statistical analyses

Data were statistically analyzed using GraphPad Prism version 9.5 (GraphPad Software, San Diego, CA, USA). Differences between groups were assessed using one-way analysis of variance, two-sample *t*-, chi-square, Fisher’s exact, and multiple-comparison tests, or Pearson correlation analysis as appropriate. Data are presented as the mean ± SD. Statistical significance was set at a *P* value < 0.05, and the significance levels were as follows:*p < 0.05, **p < 0.01, and ***p < 0.001.

## Supporting information

S1 Fig Spleen. (TIF)
