# Supplementary figures and images for: Dual role of Japanese encephalitis virus fusion loop peptide antibodies in Zika virus infection
Source: PLoS Negl Trop Dis. 2026 May 4;20(5):e0014296. doi: 10.1371/journal.pntd.0014296 (PMC13152211; doi:10.1371/journal.pntd.0014296)

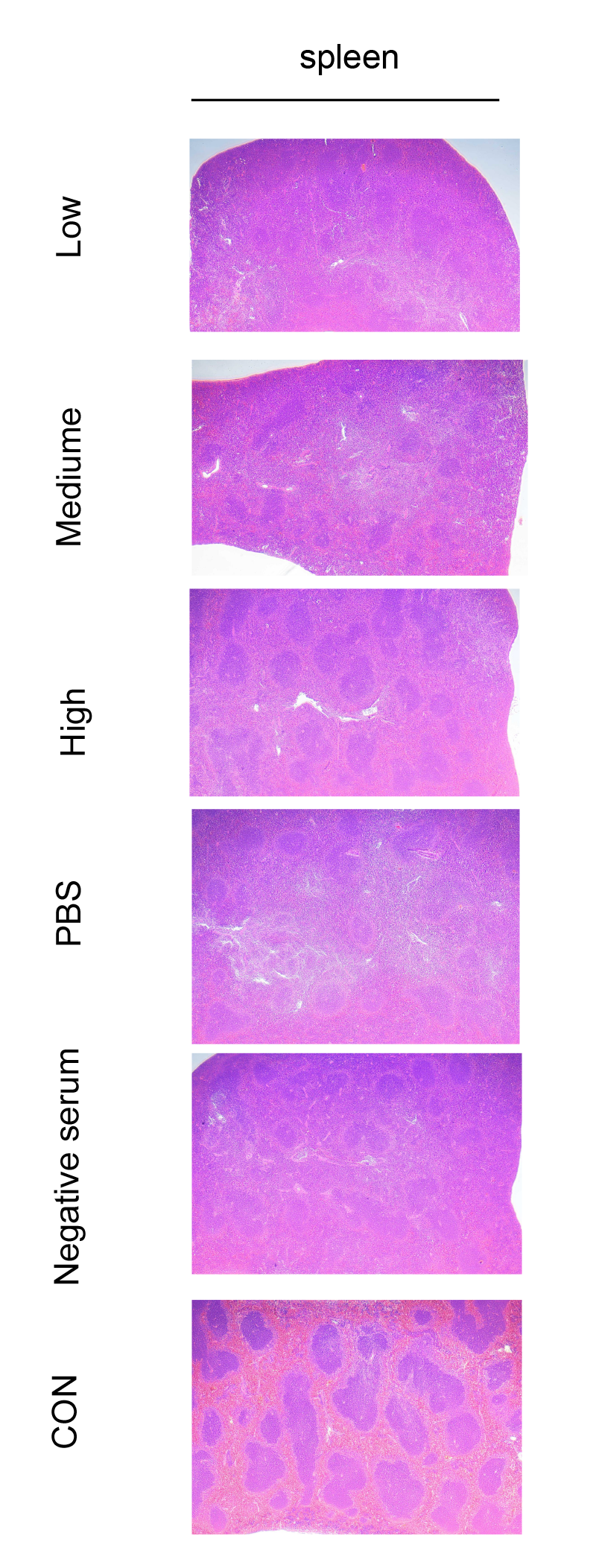

Supplement: S1 Fig — (TIF) [file pntd.0014296.s001.tif]
